# Draft Genome Sequences of Strains of *Pasteurella multocida* Isolated from the United Kingdom and the United States

**DOI:** 10.1128/genomeA.00773-13

**Published:** 2013-10-10

**Authors:** Frederick A. Lainson, Mark P. Dagleish, Michael Fontaine, Colin Bayne, J. Christopher Hodgson

**Affiliations:** Moredun Research Institute, Edinburgh, United Kingdom

## Abstract

*Pasteurella multocida* is a major pathogen of farm animals and has worldwide distribution. Here we report the draft genome sequences of four strains that were isolated from animals in the United Kingdom and the United States and represent pathogenic and commensal presentation of the bacterium.

## GENOME ANNOUNCEMENT

The Gram-negative facultatively anaerobic bacterium *Pasteurella multocida* is a pathogen that affects cattle, sheep, goats, pigs, and poultry worldwide and causes severe respiratory or septicemic infection. It is also found commonly as a commensal colonizing the upper respiratory tract in apparently healthy animals. Hotchkiss et al. (1) used multilocus sequence typing to characterize variation in isolates and relate this to host range, geographical range, and virulence. This approach delineated broad groups such as bovine, ovine, and avian isolates; however, in order to develop effective control or diagnostic strategies it is necessary to have a more detailed understanding of the genetic variability within and between groups. Here we present draft genome sequences for three ovine or bovine isolates from the United Kingdom and one bovine isolate from United States.

Genome sequencing was performed using an Illumina Solexa Genome Analyzer at the GenePool sequencing facility at the University of Edinburgh, http://genepool.bio.ed.ac.uk/. De novo assembly was carried out using Velvet version 0.7 (2), and the resulting sequence data were submitted to NCBI analysis using the Prokaryotic Genomes Automatic Annotation Pipeline (PGAAP). Details of the origins of these strains are shown in Table 1. The cumulative sequence lengths and the numbers of predicted coding sequences are consistent with those of related *P. multocida* strains sequenced previously (accession numbers NC_017764, NC_016808, NC_17027, and NC_002663). This indicates that coverage is relatively complete for each of these sequenced genomes.

**Table 1:** Details of the four sequenced *Pasteurella multocida* strains reported here

Strain name	Yr of isolation	Geographical location of isolation	Host species and tissue site of isolation	No. of contigs	Cumulative contig length (bp)	No. of proteins predicted^*a*^	% of genome with ≥98% identity to PM70^*b*^
2000	2000	United Kingdom, Scotland	Bovine, lung	261	2,210,534	2,141	93.34
P1933	2004	United States	Bovine, lung	269	2,441,686	2,441	92.65
R11F	1999	United Kingdom	Bovine, vaginal	243	2,195,634	2,129	98.58
1500E	2000	United Kingdom, Scotland	Bovine, nasal	265	2,210,576	2,148	93.37

a Number of proteins predicted in the PGAAP (Prokaryotic Genomes Automatic Annotation Pipeline) annotation process.

b Percentage length of each genome that has an identity of 98% or greater to the reference genome PM70.

Assembled contig sequences were mapped to the reference genome PM70 (3) (accession number NC_002663) using Nucmer (4). This showed extensive sequence similarity, with a high proportion of each genome mapping to PM70 with an identity of 98% or greater, as shown in Table 1. Each of these Nucmer alignments was also inspected using Mapview (4) and this showed extensive synteny, with each genome being colinear to PM70 along the majority of their length.

Despite the variation in host range and the diverse virulence characteristics among this collection of strains, their sequence variability appears to be quite localized and this may provide an insight into genetic adaptation to particular niches.

### Nucleotide sequence accession numbers.

The genome sequences for these four *P. multocida* strains have been deposited in the NCBI and assigned the following accession numbers: strain 2000, ARNW00000000; P1933, ARNY00000000; R11F, ARNZ00000000; and 1500E, AQTL00000000. In each case the version described here is NNNN00000000.1.
